# Exploration of the Structural, Electronic and Tunable Magnetic Properties of Cu_4_M (M = Sc-Ni) Clusters

**DOI:** 10.3390/ma10080946

**Published:** 2017-08-15

**Authors:** Dong Die, Ben-Xia Zheng, Xiao-Yu Kuang, Zheng-Quan Zhao, Jian-Jun Guo, Quan Du

**Affiliations:** 1 School of Science, Xihua University, Chengdu 610039, China; xhu_zbx@163.com (B.-X.Z.); dd_cpl@163.com (Z.-Q.Z.); jmst_gjj@163.com (J.-J.G.); duquanlm@163.com (Q.D.); 2 Institute of Atomic and Molecular Physics, Sichuan University, Chengdu 610065, China

**Keywords:** geometrical structure, electronic property, magnetic moment, Cu_4_M (M = Sc-Ni) cluster

## Abstract

The structural, electronic and magnetic properties of Cu_4_M (M = Sc-Ni) clusters have been studied by using density functional theory, together with an unbiased CALYPSO structure searching method. Geometry optimizations indicate that M atoms in the ground state Cu_4_M clusters favor the most highly coordinated position. The geometry of Cu_4_M clusters is similar to that of the Cu_5_ cluster. The infrared spectra, Raman spectra and photoelectron spectra are predicted and can be used to identify the ground state in the future. The relative stability and chemical activity are investigated by means of the averaged binding energy, dissociation energy and energy level gap. It is found that the dopant atoms except for Cr and Mn can enhance the stability of the host cluster. The chemical activity of all Cu_4_M clusters is lower than that of Cu_5_ cluster whose energy level gap is in agreement with available experimental finding. The magnetism calculations show that the total magnetic moment of Cu_4_M cluster mainly come from M atom and vary from 1 to 5 *μ*_B_ by substituting a Cu atom in Cu_5_ cluster with different transition-metal atoms.

## 1. Introduction

The binary alloy clusters have been investigated widely during the past several decades [1,2,3,4,5,6,7,8,9,10,11,12,13,14,15,16,17,18,19,20,21,22,23,24,25,26,27,28,29,30,31,32,33,34]. Experimental and theoretical research has manifested that the introduction of a dopant atom into a small cluster can considerably change the nature of the host cluster. Copper clusters doped with an impurity atom have been actively pursued to tailor the desired structural, electronic, magnetic and optical properties for potential applications in solid state chemistry, materials science, nanotechnology and microelectronics [35,36,37,38,39,40,41,42,43,44,45,46,47]. For example, the bimetallic Cu*_n_*Pd*_m_* (m+n≤6) clusters are more stable than the monometallic particles with the same size [35]. The presence of Cr dopant obviously enhances the stability of Cu*_n_*Cr (*n* = 9–16) in comparison to that of pure counterparts [36]. The bond stiffness of the copper cluster are decreased after doping with Pd atoms. The most stable Cu_7_Sc, Cu_15_Sc and Cu_16_Sc clusters could be regarded as a σ-aromatic species, a superatom and the germ of a crystallization process, respectively [37,38,39]. The Ti- and V-doping dramatically improves the adsorption of copper clusters on NO molecules, but it does not affect the O_2_ adsorption probability significantly [40]. The Cu-Fe icosahedral nano-clusters exhibit larger magnetic moments than the Fe thin films and bulk systems [41]. The Cu*_n_*Se clusters are the perfect candidate for renewable energy sources in the photocatalysis field [42]. Recently, the Cu_2_, Cu_6_ and Cu_12_ clusters doped with various atoms have received particular interest owing to their unique physical and chemical properties [48,49,50]. It was shown that, among all Cu_2_X (X = Sc-Zn) clusters, the Cu_2_Ti is found to have the highest ability of the dissociation absorption of H_2_ moleculaes [48]. The Cu_6_Co cluster is the best catalyst for the water–gas–shift reaction (CO + H_2_O→C_2_O + H_2_, $\Delta H_{25{}^{\circ}C}=-41\;\mathrm{kJ}/\mathrm{mol}$ ) [49]. The doping of single 3*d* transition metal atoms would overcome the problem of stabilization at the noble Cu_12_ icosahedra [50]. As far as we know, however, there is relatively few systematic work concerning the doped Cu_4_ clusters. On the other hand, it is well known that the bimetallic clusters often have intriguing properties, which should be very different from those of the bulk materials or atoms, in virtue of the so-called surface and size effects. Therefore, in this paper, the structural, electronic, and magnetic properties of small Cu_4_M (M = Sc-Ni) clusters are studied systematically by means of density functional theory (DFT). It is hoped that this work could provide valuable information to realize the influence of dopant atom and would be of help to chemists, especially those designing new nanomaterials.

## 2. Computational Methods

Geometry optimizations and vibrational frequency computations were performed by using B3LYP, (Becke A. D., Kingston, ON, Canada) hybrid exchange–correlation functional in conjunction with an effective core potential LanL2DZ basis sets, as implemented in the GAUSSIAN09 program package (Frisch, M. J. et al., Wallingford, KY, USA) [51,52,53,54,55]. The default convergence thresholds are used in all computations. One hundred and twenty initial configurations of each Cu_4_M cluster were generated by the CALYPSO soft (Ma Y.M., Changchun, Jilin, China) [56]. A local version of particle swarm optimization algorithm is employed to utilize a fine exploration of potential energy surface. Similar structures can be distinguished by the bond characterization matrix. Due to the spin polarization, every initial configuration was optimized at various possible spin states. All low-energy isomers obtained at B3LYP/LanL2DZ level were also calculated at the BLYP/6-311+G(d) level. The accuracy of the computational method has been checked by calculations on Cu_2_, Ti_2_, V_2_, Cr_2_, Fe_2_ and Ni_2_ dimers. The calculated results summarized in Table 1 are in good agreement with available experimental findings.

## 3. Results and Discussion

### 3.1. Geometrical Structures and Vibrational Spectra

**Figure 1:**
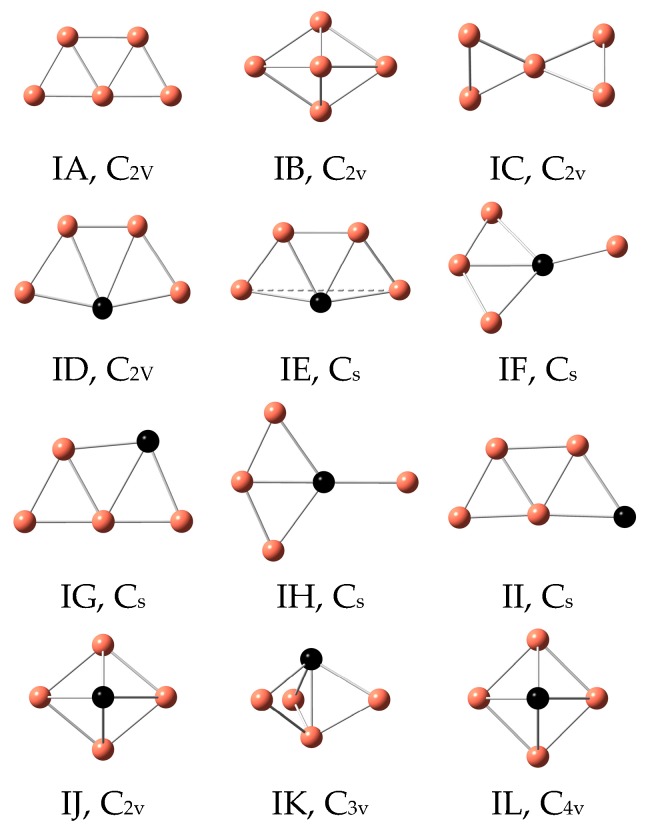
The ground state and low-lying isomers of Cu_4_M (M = Sc-Cu) clusters.

In order to examine the effects of M (M = Sc-Ni) atoms on copper clusters, structural searches of the Cu_5_ cluster were carried out firstly using the abovementioned functional and basis sets. The ground state and low-lying structures of Cu_5_ and Cu_4_M clusters are plotted in Figure 1. These structures are denoted by IA, IB, IC, etc. Their energy difference (ΔE) compared to each of the ground state and spin multiplicity (SM) are listed in Table 2. Some physical parameters of the lowest energy Cu_5_ and Cu_4_M clusters are collected in Table 3.

The results of two theoretical levels show that the ground state structure of Cu_5_ clusters is an isosceles trapezium. This is consistent with previous reports [57]. The most stable structure of Cu_4_Sc cluster is planar ID isomer for B3LYP/LanL2DZ level and three-dimensional (3D) IE isomer for BLYP/6-311+G(d) level. The former at BLYP/6-311+G(d) level is unstable and almost degenerate with the latter, which is very close to the planar configuration. The latter at B3LYP/LanL2DZ level is 0.07 eV higher in energy than the former. The IF isomer of Cu_4_Sc cluster is obtained by distorting the geometry starting from C_2v_ to C_s_ symmetry. The lowest energy structure of Cu_4_Ti cluster is 3D IE isomer. The Cu–Ti–Cu bond angle is about 158°. Its triplet spin state is lower in energy than other spin state. It is worth mentioning that there is a T_d_ configuration for Au_4_Ti cluster [63], but this configuration for Cu_4_Ti cluster turns into an IF isomer. In the case of Cu_4_M (M = V-Fe and Ni) clusters, all of the ground state structures exhibit a planar structure similar to the most stable Cu_5_ cluster. Other planar and 3D isomers are found as the low-lying isomers. The optimized results for Cu_4_Cr cluster reveal that the quintet spin state is more stable than the triplet and septet spin states. In addition, the 3D IE configuration becomes a planar ID structure after geometric optimization and the IF structure does not exist for Cu_4_M (M = V-Fe and Ni) clusters. The most stable structure of Cu_4_Co cluster resembles the ground state of Cu_4_Ti cluster. The Cu–Co–Cu bond angle is 168°. The ID isomer for Cu_4_Co cluster is obtained only at the B3LYP/LanL2DZ level. The other 3D isomers of Cu_4_Co cluster, such as trigonal bipyramid and tetragonal pyramid, etc., are less stable than the ground state. From the optimized results, it is obvious that the M (M = Sc-Ni) atoms in the lowest energy Cu_4_M clusters tend to occupy the most highly coordinated position. This phenomenon is in accordance with the principle of maximum overlap in molecular orbital theory. Next, we will discuss the results based on the B3LYP/LanL2DZ level.

**Figure 2:**
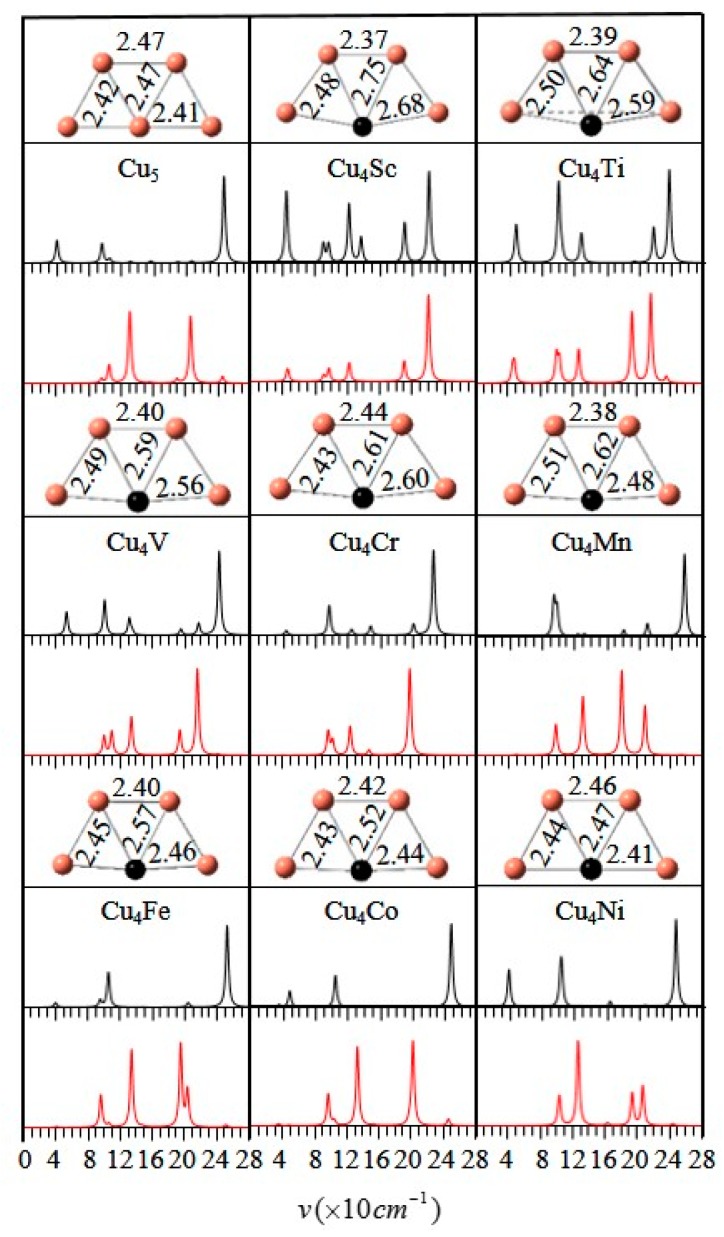
Vibrational (black) and Raman (red) spectra of the ground state Cu_4_M (M = Sc-Cu) clusters.

The comparison of calculated spectra and experimental spectra is an effective method to determine the structures of small isolated metal clusters. As a result, the vibrational and Raman spectra of the ground state Cu_5_ and Cu_4_M (M = Sc-Ni) clusters are computed and displayed in Figure 2. The two types of spectra belong to absorption and scattering spectra, respectively. The fundamental frequencies of the vibrational spectra and Raman spectra for all isomers are in the range of 30–260 and 40–240 cm^−1^. For the same vibrational frequency, if a weak peak occurs in the vibrational spectrum, then a strong peak will exist in the Raman spectrum. The most intense peak in all vibrational spectra of Cu_4_M is related to the Cu-M-Cu antisymmetric stretching vibration. The substitution of Sc, Ti and V atoms for Cu atom has a great influence on the spectra of the host cluster. The vibrational spectra of Cu_4_Co and Cu_5_ clusters look very similar, whereas the distance between the second peak and the third peak of the latter is 8.4 cm^−1^ greater than that of the former. These spectra can indeed be used as the fingerprint signals to identify their geometrical structures.

### 3.2. Electronic Properties

**Figure 3:**
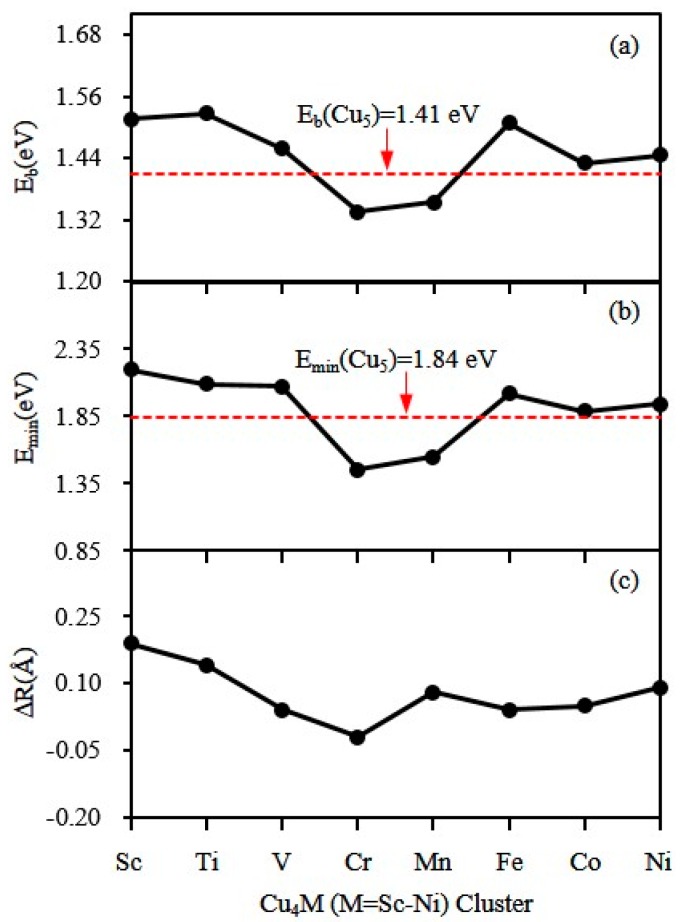
The atomic averaged binding energy (**a**), the minimum DE (**b**) and the overlap of orbital radius for the ground-state Cu_4_M (M = Sc-Cu) clusters (**c**).

Binding energy is an important parameter to reflect the relative thermal stability of clusters. Some properties of materials can be estimated by using the data of binding energy. The atomic averaged binding energies ($E_{b}$) of the Cu_4_M and Cu_5_ clusters are calculated by the following formula:

(1) $$E_{b}(Cu_{4}M)=\left[4E(Cu)+E(M)-E(Cu_{4}M)\right]/5,$$

(2) $$E_{b}(Cu_{5})=\left[5E(Cu)-E(Cu_{5})\right]/5,$$

where $E(Cu_{4}M)$, E(Cu), E(M) and $E(Cu_{5})$ denote the energy of Cu_4_M cluster, a Cu atom, a dopant atom and Cu_5_ cluster. The calculated $E_{b}$ for the most stable Cu_4_M and Cu_5_ clusters is shown in Figure 3a. The $E_{b}$ of Cu_4_M clusters is bigger for M = Sc, Ti, V, Fe, Co and Ni and smaller for M = Cr and Mn than that of Cu_5_ cluster. That is to say, apart from Cr and Mn atoms, the replacement of a Cu atom by single M (M = Sc, Ti, V, Fe, Co and Ni) atom increases the stability of the host clusters. At the same time, the thermal stability of clusters can also be analyzed by the minimum dissociation energy, which involves the dissociation channels below:

(3) $$Cu_{5}\to \;Cu_{m}+\;Cu_{5-m},$$

(4) $$Cu_{4}M\to \;Cu_{m}+\;Cu_{4-m}M,$$

where *m* is less than or equal to 4. The corresponding dissociation energy (DE) is calculated as follows:

(5) $$DE_{m}(Cu_{5})=E(Cu_{5-m})+E(Cu_{m})-E(Cu_{5}),$$

(6) $$DE_{m}(Cu_{4}M)=E(Cu_{4-m}M)+E(Cu_{m})-E(Cu_{4}M),$$

where *E* denotes the energy of the corresponding cluster or atom. The DEs of the lowest energy Cu_5_ and Cu_4_M clusters for the distinct dissociation channels have been given in Table 4. The minimum DEs of Cu_5_ and Cu_4_M have been shown as a function of dopant atoms in Figure 3b. This is also in line with the above analysis based on averaged binding energy. In order to understand $E_{b}$ and DE further, the overlap of orbital radius (ΔR) is calculated using the following formula:

(7) $$\Delta R=R_{Cu}+R_{M}-R_{\bar{V}},$$

where $R_{Cu}$, $R_{M}$ and $R_{\bar{V}}$ are the radius of Cu and M atoms and the average coordination bond length of the M atom in the Cu_4_M cluster. The calculated ΔR is displayed in Figure 3c. The overlap of the orbital radius of Fe atom and Cu atoms is slightly smaller than the one we expect. Overall, the ΔR, DE and $E_{b}$ basically maintain a consistent change. This means that the binding energy is closely related to the overlap of the electron cloud.

**Figure 4:**
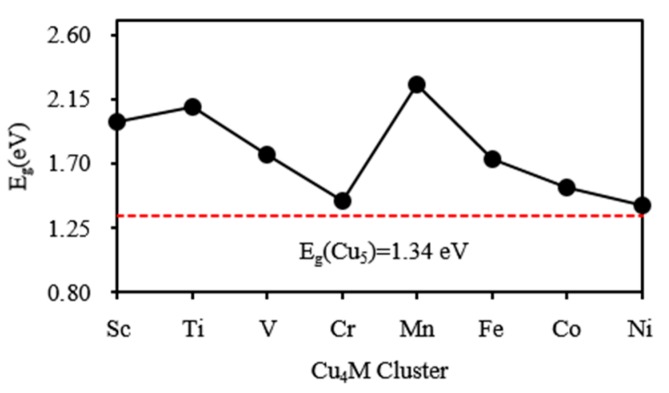
The HOMO-LUMO energy gaps of the ground state Cu_4_M (M = Sc-Cu) clusters.

**Figure 5:**
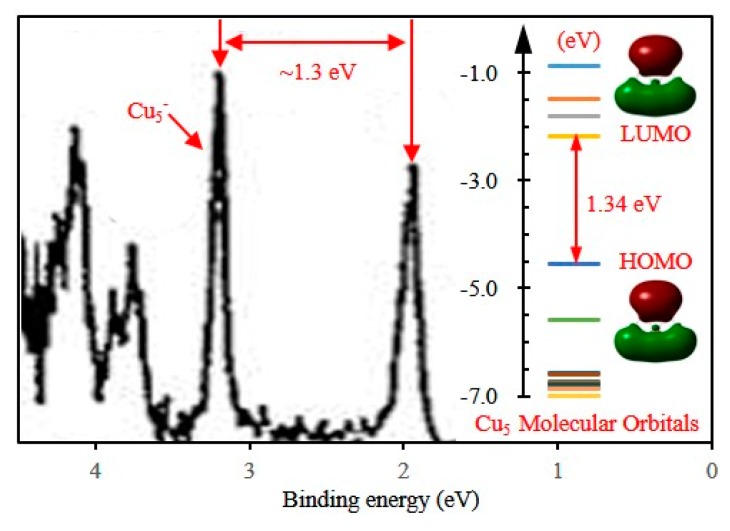
The experimental PES spectrum of Cu_5_^−^ cluster, which is cited from Ref. [64].

**Figure 6:**
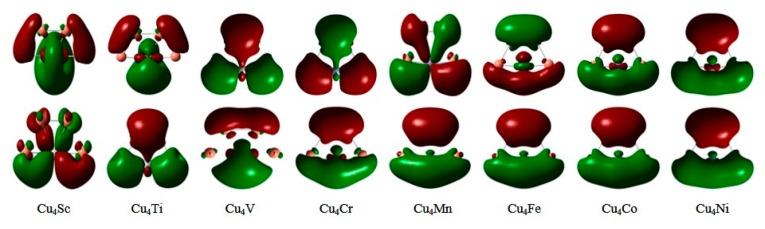
The LUMO (**first row**) and HOMO (**second row**) molecular orbitals of the ground state Cu_4_M (M = Sc-Ni) clusters.

The energy gap (*E*_g_) between the highest occupied molecular orbital (HOMO) and lowest unoccupied molecular orbital (LUMO) is a fairly important quantity that reflects chemical activity of small clusters. The cluster with a large energy gap usually has high chemical stability. For the ground state Cu_5_ and Cu_4_M (M = Sc-Ni) clusters, the HOMO-LUMO energy gaps are calculated and shown in Figure 4. The Cu_5_ cluster with C_2v_ symmetry has an energy gap of 1.34 eV. The energy gap of neutral Cu_5_ cluster can be estimated experimentally by photoelectron spectrum (PES) spectra of the corresponding anionic cluster. The experimental PES spectra of Cu_5_^−^ was reported by Cha et al. [64] and is shown in Figure 5. The energy difference between the first and second peaks in PES spectra of anionic clusters is an approximate measure of the HOMO-LUMO gap of the corresponding neutral clusters. The energy gap of Cu_5_ clusters has been measured and is 1.30 eV. The measured value is in agreement with our calculated result. The energy gaps of Cu_4_M clusters whose LUMO and HOMO diagram are shown in Figure 6 are bigger than that of Cu_5_ clusters. The substitution of Cu atom with M (M = Sc-Ni) atom improves the chemical stability of the host cluster. The Cu_4_Ti and Cu_4_Mn clusters have a large energy gap relative to the neighbouring clusters. The large energy gaps can be interpreted by an eight electron rule for Cu_4_Ti and a half-filled *d* orbital for Cu_4_Mn. The two clusters presumably are less reactive and should be useful as a building block for constructing the cluster-assembled materials.

**Figure 7:**
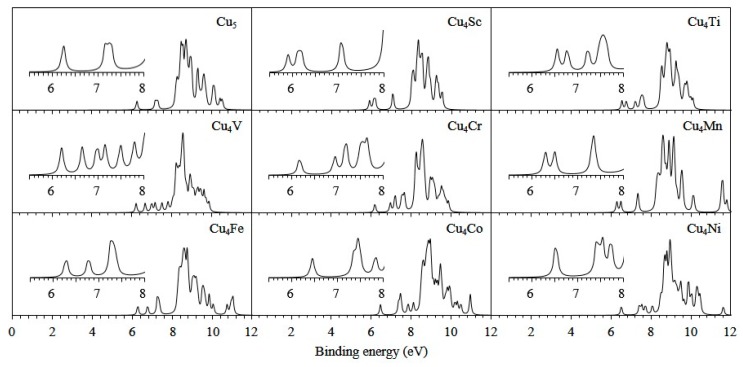
Simulated PES of the ground state Cu_4_M (M = Sc-Cu) clusters.

The vertical ionization potential (VIP) and electron affinity (EA) can reflect the ability of the cluster to lose electrons and capture electrons and are defined as follows:

(8) $$\begin{array}{l} VIP=E({\mathrm{cluster}}^{+})-E(\mathrm{cluster})\\ EA=E(\mathrm{cluster})-E({\mathrm{cluster}}^{-}) \end{array},$$

where $E({\mathrm{cluster}}^{+})$ and $E({\mathrm{cluster}}^{-})$ are the single point energies of the cationic and anionic clusters in the corresponding neutral geometry. For the most stable Cu_5_ and Cu_4_M clusters, the calculated first VIP, EA and the available experimental data are given in Table 5. The calculated VIP and EA of Cu_5_ are in agreement with the experimental findings [65,66]. Thereby, the reliability of the current calculation method is again demonstrated. In all clusters, the VIP of Cu_4_Ti is the largest and the EA of Cu_4_Mn is the smallest. This may be attributed to the fact that the Cu_4_Ti with eight valence electrons is not easy to lose electrons and the half-filled 3*d* orbitals of Mn atoms in Cu_4_Mn are not readily accessible to electrons. The photoelectron spectroscopy (PES) of clusters can be obtained by VIP and HOMO. The simulated PES of the lowest energy Cu_5_ and Cu_4_M clusters are obtained by adding the occupied orbital energy relative to the HOMO to the VIP and fitting them with a broadening factor of 0.1 eV, as plotted in Figure 7. An intense band at 5.5–12 eV are apparent in the PES of all clusters. The doping atoms significantly change the PES of the host cluster, especially in the range of 6–8 eV. It should be pointed out that the Cu_4_Co and Cu_5_ clusters have similar vibrational spectra but different PES, which can be employed to identify their geometrical structures.

### 3.3. Magnetic Properties

**Figure 8:**
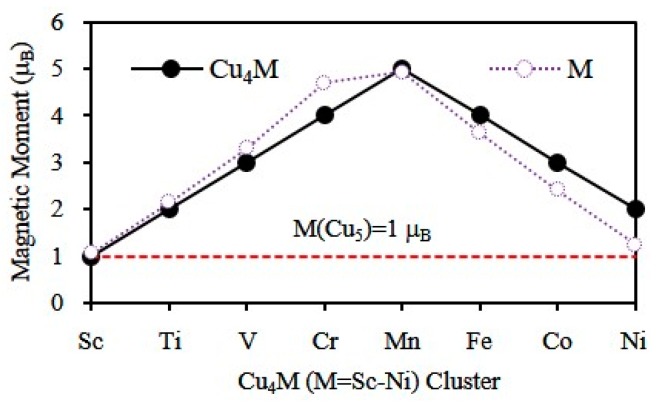
Total magnetic moment of the ground state Cu_4_M (M = Sc-Cu) clusters and local magnetic moment on the dopant atoms.

**Figure 9:**
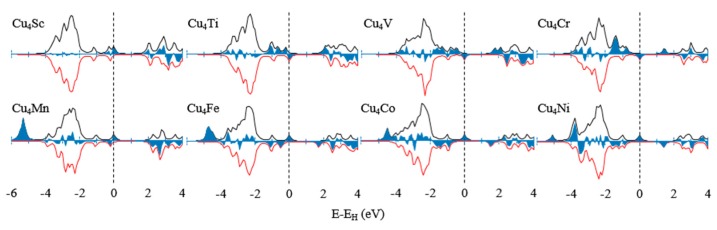
The SDOS of ground state Cu_4_M (M = Sc-Ni) clusters. A broadening factor δ = 0.1 eV is used. Spin up (positive) and spin down (negative) densities are given in each case. The blue part is the density difference (spin up minus spin down). The dashed line indicates the location of the HOMO level.

The DFT provides a powerful tool for the study of the magnetic properties of metal clusters. The total magnetic moment of cluster chiefly comprises the orbital and spin magnetic moments of electron. The orbital magnetic moment of an electron is much smaller than its spin magnetic moment and, hence, the magnetic moments of clusters are mainly from spin magnetic moments. The total magnetic moment of the ground state Cu_5_ and Cu_4_M (M = Sc-Ni) clusters has been calculated and is shown in Figure 8. The magnetic moment of Cu_4_Mn (5 *μ*_B_) is the largest in all doped clusters. The magnetic moment of other Cu_4_M clusters is 1, 2, 3, 4, 4, 3 and 2 *μ*_B_ for M = Sc, Ti, V, Cr, Fe, Co and Ni, respectively. Interestingly, the magnetic moment of Cu_4_M clusters is equal to that of free M atoms, except for M = Cr. This exception can be attributed to the opened 4s shell of Cr atom, while other atoms have a closed 4s shell. The substitution of a Cu atom by a single M (M = Ti-Ni) atom can enhance the magnetism of the host clusters. The various magnetic moments hint that the Cu_4_M clusters have potential utility in new nanomaterials with tunable magnetic moments. As an effort to explain the magnetism, Figure 9 displays the spin density of states (SDOS) for the global minimum structures of Cu_4_M clusters. All of the ground state clusters have a strong band between −4 and −2 eV, which consists chiefly of the valence *s* and *d* orbitals of the constituent atoms. It is clear from this figure that the magnetic moment of Cu_4_M (M = Sc-Cr) clusters is generated by the electrons near the HOMO. The magnetic moment of Cu_4_M (M = Mn-Ni) largely derives from the energy level far away from HOMO. This implies that the Cu_4_Mn, Cu_4_Fe, Cu_4_Co and Cu_4_Ni clusters may be a hard magnetic nanomaterial.

**Figure 10:**
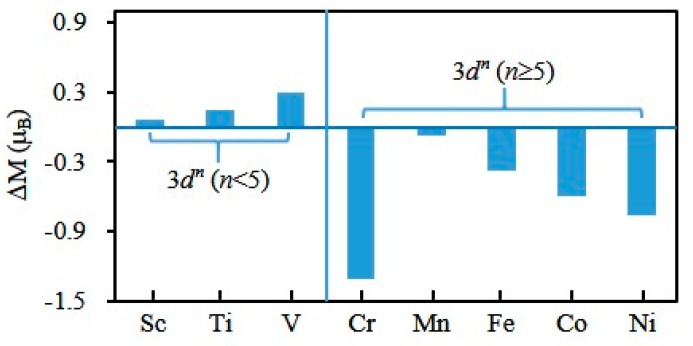
The magnetic moment of the free M atom as the reference point, the change of magnetic moments of the M atom in Cu_4_M (M = Sc-Ni) clusters.

To explore the magnetic properties further, we have executed the natural bond orbital analysis [67] for the most stable Cu_4_M (M = Sc-Ni) clusters. The local magnetic moments on M atom is 1.06, 2.14, 3.29, 4.69, 4.93, 3.62, 2.41 and 1.24 *μ*_B_ for M = Sc, Ti, V, Cr, Mn, Fe, Co and Ni atoms, as shown in Figure 8. The total magnetic moment of Cu_4_M clusters is mainly localized on the M atom. The magnetic moment yielded by the copper atom is small in the total magnetic moment. Furthermore, Cu atoms in Cu_4_M (M = Sc-Cr) clusters exhibit an antiferromagetic alignment with respect to the M atom’s magnetic moment. Compared with the free atom, the magnetic moment of the M atom in the copper cluster increases for M = Sc, Ti and V atoms and reduces for M = Cr, Mn, Fe, Co and Ni atoms, as displayed in Figure 10. This may be because the number of electrons in the 3*d* shell of dopant atom is less than 5 from Sc to V atoms and is greater than or equal to 5 (≥5) from Cr to Ni atoms.

**Figure 11:**
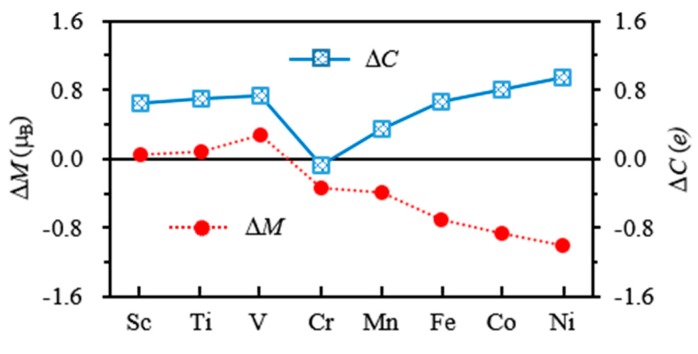
The change of magnetic moment (Δ*M*) and the charge transfer (Δ*C*) of 3*d* orbital of M atom in Cu_4_M (M = Sc-Ni) clusters.

The charge and local magnetic moment on 3*d*, 4*s*, 4*p* and 5*p* shells of M atoms in Cu_4_M (M = Sc-Cr) clusters are listed in Table 5. It can be seen from Table 5 that the magnetic moment of M atom mainly comes from its 3*d* shell. The 4*s*, 4*p* and 5*p* shells bring a small amount of magnetic moments. In contrast to the isolated atoms, the charge on the 3*d* shell of M atoms except Cr increases 0.35–0.95 e. The charge on 4*s* shell of all dopant atoms decreases by 0.47–1.41 e. In addition, the 4*p* and 5*p* shells of M atoms in Cu_4_M cluster are also found to have a number of charges. The distribution of shell charge shows that some electrons of the M atom are transferred from 4*s* shell to 3*d*, 4*p* and 5*p* shells. At the same time, an interatomic charge transfer takes place in the Cu_4_M clusters. According to our calculations, 0.1–0.47 e transfer from Sc, V and Cr to Cu atoms; however, 0.01–0.26 e transfer from Cu to Ti, Mn, Fe, Co and Ni atoms. The charge transfer suggests that the M atom in Cu_4_M clusters has an orbital hybridization among *s*, *p* and *d* shells. If ΔM and ΔC denote the changes of magnetic moments and charge of 3*d* shells of M atoms, we find that the |ΔM| increases with the |ΔC| increasing, as displayed in Figure 11. It can be deduced from this figure that the charge transfer should be the primary reason for the change of the magnetic moment of M atoms in Cu_4_M clusters.

## 4. Conclusions

The structural, electronic and magnetic properties of Cu_4_M (M = Sc-Ni) clusters have been investigated by the CALYPSO structure searching method and density functional theory. The structural searches reveal that M atoms in the most stable Cu_4_M clusters favor the most highly coordinated position. The structure of Cu_4_M clusters resembles that of Cu_5_ cluster. The infrared spectra, Raman spectra and PES are given to identify the ground state. Research of electronic properties shows that the M atoms in Cu_4_M (M = Sc-V and Fe-Ni) clusters can improve the stability of the host cluster. The energy gap of all Cu_4_M clusters is bigger than that of Cu_5_ cluster. The magnetism analyses indicate that the 3*d* transition-metal atom in the Cu_4_M cluster carries most of the total magnetic moment. The change of magnetic moment is closely related to the charge transfer.

## Figures and Tables

**Table 1:** The geometries and electronic properties of Cu_2_, Ti_2_, V_2_, Cr_2_ and Fe_2_ dimers.

Dimer	Functional/Basis Set	*R* (Å)		D_e_ (eV)		f (cm^−1^)		VIP (eV)		EA (eV)	
		Calc.	Expt.	Calc.	Expt.	Calc.	Expt.	Calc.	Expt.	Calc.	Expt.
Cu_2_	B3LYP/LanL2DZ	2.26	2.22 ^a^	2.02	2.01 ^a^	256	264 ^a^	7.99	7.90 ^a^	0.63	0.83 ^a^
	Blyp/6-311+G(d)	2.27		2.01		244		8.20		0.83	
Ti_2_	B3LYP/LanL2DZ	1.91	1.94 ^b^	1.42	1.54 ± 0.19 ^b^						
V_2_	B3LYP/LanL2DZ	1.75	1.77 ^c^	1.94	2.47 ± 0.22 ^c^			6.39	6.35 ^c^		
Cr_2_	B3LYP/LanL2DZ							6.22	6.4 ± 0.2 ^d^		
Fe_2_	B3LYP/LanL2DZ	2.15	2.02 ^e^	1.28	1.30 ^e^			6.24	6.30 ^e^		
Ni_2_	B3LYP/LanL2DZ	2.38	2.20 ^f^	1.59	2.06 ^f^						

^a^ Ref. [57], ^b^ Ref. [58], ^c^ Ref. [59], ^d^ Ref. [60], ^e^ Ref. [61], ^f^ Ref. [62].

**Table 2:** The lower energy isomer (LI), ΔE and spin multiplicity (SM) of Cu_5_ and Cu_4_M (M = Sc-Ni) clusters at the B3LYP/LanL2DZ and Blyp/6-311+G(d) levels.

Clusters	B3LYP/LanL2DZ			Blyp/6-311+G(d)		
	LI	ΔE (eV)	SM	LI	ΔE (eV)	SM
Cu_5_	IA	0	2	IA	0	2
	IB	0.54	2	IB	0.44	2
	IC	0.88	2	IC	0.91	2
Cu_4_Sc	ID	0	2	IE	0	2
	IE	0.07	2	IF	0.07	2
	IF	0.21	2	IJ	0.09	2
Cu_4_Ti	IE	0	3	IE	0	3
	ID	0.15	3	ID	0.15	3
	IH	0.22	3	IL	0.28	3
Cu_4_V	ID	0	4	ID	0	4
	IG	0.36	4	IE	0.28	4
	IL	0.43	4	IK	0.49	4
Cu_4_Cr	ID	0	5	ID	0	5
	IG	0.13	7	IG	0.36	5
	II	0.24	7	IJ	0.43	5
Cu_4_Mn	ID	0	6	ID	0	6
	IG	0.16	6	IG	0.23	6
	IH	0.24	6	IH	0.26	6
Cu_4_Fe	ID	0	5	ID	0	5
	IG	0.11	5	IG	0.18	5
	II	0.25	5	IJ	0.33	5
Cu_4_Co	IE	0	4	IE	0	4
	ID	0.01	4	IG	0.16	4
	IG	0.18	2	II	0.31	4
Cu_4_Ni	ID	0	3	ID	0	3
	IG	0.09	3	IG	0.10	3
	II	0.31	3	IL	0.12	3

**Table 3:** The dipole moment (*μ*), polarizability ($a_{xx},\;a_{yy},\;a_{\mathrm{zz}},\;\bar{a}$) and zero-point energy (ZPE) of the most stable Cu_5_ and Cu*_n_*M (M = Sc-Ni) clusters and average coordination bond length ($R_{\bar{V}}$) for M atoms.

Clusters	*µ* (D)	$a_{xx}$ (a.u.)	$a_{yy}$ (a.u.)	$a_{zz}$ (a.u.)	$\bar{a}$ (a.u.)	ZPE (J/mol)	$R_{\bar{V}}$ (Å)
Cu_5_	0.01	282.6	199.3	105.0	195.6	7265.7	
Cu_4_Sc	2.22	314.1	223.6	182.7	240.1	7020.5	2.72
Cu_4_Ti	1.54	128.1	206.6	304.1	212.9	7204.7	2.61
Cu_4_V	1.07	299.2	200.6	118.6	206.1	7383.0	2.58
Cu_4_Cr	0.83	299.3	196.2	115.6	203.7	7077.0	2.60
Cu_4_Mn	0.56	116.0	286.7	217.2	206.6	7148.5	2.55
Cu_4_Fe	0.37	110.7	283.8	206.7	200.4	7319.2	2.51
Cu_4_Co	0.27	108.1	202.5	283.1	197.9	7315.1	2.48
Cu_4_Ni	0.08	283.2	201.9	106.4	197.2	7532.0	2.44

**Table 4:** The dissociation energy (DE) of Cu_5_ and Cu_4_M (M = Sc-Ni) clusters for the distinct dissociation channels.

Dissociation Channels		DE (eV)			
		*m* = 1	*m* = 2	*m* = 3	*m* = 4
Cu_5_ = Cu*_m_* + Cu_5−*m*_		1.84	2.03		
Cu_4_Sc= Cu*_m_*+ Cu_4−*m*_Sc		2.23	2.20	3.10	2.36
Cu_4_Ti = Cu*_m_*+ Cu_4−*m*_Ti		2.20	2.09	2.92	2.41
Cu_4_V = Cu*_m_* + Cu_4−*m*_V		2.12	2.10	2.81	2.07
Cu_4_Cr = Cu*_m_* + Cu_4−*m*_Cr		1.86	2.03	2.17	1.45
Cu_4_Mn = Cu*_m_* + Cu_4−*m*_Mn		2.18	1.73	2.78	1.55
Cu_4_Fe = Cu*_m_* + Cu_4−*m*_Fe		2.02	2.11	2.47	2.32
Cu_4_Co = Cu*_m_* + Cu_4−*m*_Co		1.89	2.08	2.28	1.93
Cu_4_Ni = Cu*_m_* + Cu_4−*m*_Ni		1.94	2.11	2.34	2.00

**Table 5:** VIP and EA of the ground state Cu_4_M (M = Sc-Ni) and Cu_5_ clusters, and the charge (Q) and local magnetic moment (M) of 3*d*, 4*s*, 4*p*, and 5*p* states for the M atom in the ground state Cu_4_M clusters.

Clusters	VIP (eV)	EA (eV)	M-3*d*		M-4*s*		M-4*p*		M-5*p*	
			Q (e)	M (*μ_B_*)	Q (e)	M (*μ_B_*)	Q (e)	M (*μ_B_*)	Q (e)	M (*μ_B_*)
Cu_5_	6.24 (6.30)	1.70 (1.82)								
Cu_4_Sc	5.89	1.08	1.65	0.96	0.63	0.09	0.04	0	0.21	0.02
Cu_4_Ti	6.55	1.23	2.70	2.08	0.60	0.04	0.56	0.02	0.30	0
Cu_4_V	6.20	1.41	3.73	3.27	0.59	0.01	0.55	0.01	0	0
Cu_4_Cr	6.17	1.61	4.92	4.66	0.55	0.03	0.43	0	0	0
Cu_4_Mn	6.29	0.95	5.35	4.61	0.89	0.21	0.77	0.11	0	0
Cu_4_Fe	6.28	1.10	6.66	3.30	0.86	0.20	0.64	0.12	0	0
Cu_4_Co	6.45	1.63	7.8	2.13	0.81	0.17	0.57	0.11	0	0
Cu_4_Ni	6.52	1.57	8.95	0.99	0.78	0.14	0.22	0.08	0.31	0.03
